# Mitovesicles secreted into the extracellular space of brains with mitochondrial dysfunction impair synaptic plasticity

**DOI:** 10.1186/s13024-024-00721-z

**Published:** 2024-04-14

**Authors:** Pasquale D’Acunzo, Elentina K. Argyrousi, Jonathan M. Ungania, Yohan Kim, Steven DeRosa, Monika Pawlik, Chris N. Goulbourne, Ottavio Arancio, Efrat Levy

**Affiliations:** 1 Center for Dementia Research, Nathan S. Kline Institute for Psychiatric Research, 10962 Orangeburg, NY USA; 2 Department of Psychiatry, New York University Grossman School of Medicine, 10016 New York, NY USA; 3 Department of Pathology and Cell Biology, Taub Institute for Research on Alzheimer’s Disease and the Aging Brain, Columbia University, 10027 New York, NY USA; 4 Department of Medicine, Columbia University, 10027 New York, NY USA; 5 Department of Biochemistry & Molecular Pharmacology, New York University Grossman School of Medicine, 10027 New York, NY USA; 6 NYU Neuroscience Institute, New York University Grossman School of Medicine, 10016 New York, NY USA

**Keywords:** Alzheimer’s disease, Down syndrome, Extracellular vesicle, Exosome, Long-term potentiation, MAO-B, Microvesicle, Mitochondria, Mitovesicle, Neurodegenerative disease

## Abstract

**Background:**

Hypometabolism tied to mitochondrial dysfunction occurs in the aging brain and in neurodegenerative disorders, including in Alzheimer’s disease, in Down syndrome, and in mouse models of these conditions. We have previously shown that mitovesicles, small extracellular vesicles (EVs) of mitochondrial origin, are altered in content and abundance in multiple brain conditions characterized by mitochondrial dysfunction. However, given their recent discovery, it is yet to be explored what mitovesicles regulate and modify, both under physiological conditions and in the diseased brain. In this study, we investigated the effects of mitovesicles on synaptic function, and the molecular players involved.

**Methods:**

Hippocampal slices from wild-type mice were perfused with the three known types of EVs, mitovesicles, microvesicles, or exosomes, isolated from the brain of a mouse model of Down syndrome or of a diploid control and long-term potentiation (LTP) recorded. The role of the monoamine oxidases type B (MAO-B) and type A (MAO-A) in mitovesicle-driven LTP impairments was addressed by treatment of mitovesicles with the irreversible MAO inhibitors pargyline and clorgiline prior to perfusion of the hippocampal slices.

**Results:**

Mitovesicles from the brain of the Down syndrome model reduced LTP within minutes of mitovesicle addition. Mitovesicles isolated from control brains did not trigger electrophysiological effects, nor did other types of brain EVs (microvesicles and exosomes) from any genotype tested. Depleting mitovesicles of their MAO-B, but not MAO-A, activity eliminated their ability to alter LTP.

**Conclusions:**

Mitovesicle impairment of LTP is a previously undescribed paracrine-like mechanism by which EVs modulate synaptic activity, demonstrating that mitovesicles are active participants in the propagation of cellular and functional homeostatic changes in the context of neurodegenerative disorders.

**Supplementary Information:**

The online version contains supplementary material available at 10.1186/s13024-024-00721-z.

## Background

Extracellular vesicles (EVs) are nanometer-sized vesicles that are constitutively released into the extracellular environment by all cell types [1]. EVs are involved in mechanisms that are crucial for cellular homeostasis and for pathogenic conditions, including waste disposal, cell-to-cell communication, immunity, oxidative stress regulation, and gene expression control via miRNA transfer [1]. Although highly heterogeneous, small EVs were divided into two main groups– plasma membrane-derived microvesicles and late endosome-derived exosomes– according to their intracellular origin, size, presence/absence of specific constituents, and functions [2].

To study brain EVs and their content under normal conditions and the contributions of specific EV subtypes to brain pathology, our laboratory developed a method to isolate and characterize different EV subpopulations from postmortem brain tissue [2–9]. Using this method we found changes in the amount and content of EVs during aging [5], in mice chronically exposed to cocaine [6, 9], and in neurodegenerative and neurodevelopmental disorders such as spinocerebellar ataxia [7], Alzheimer’s disease (AD) [3], and Down syndrome (DS) [2, 4, 10]. Furthermore, fractionation of EV subtypes with a high-resolution density gradient revealed the existence of a previously unidentified type of small EVs in the brain of wild-type mice, later confirmed in human tissue [2, 8]. These EVs contain a limited, highly specialized set of mitochondrial constituents, including mitochondria-specific proteins found in the mitochondrial outer membrane, mitochondrial inner membrane, and mitochondrial matrix, such as the voltage-dependent anion channel (VDAC), the cytochrome c oxidase subunit 4 (COX-IV), and the pyruvate dehydrogenase E1 component subunit alpha (PDH-E1α), respectively [2, 8]. Under cryogenic electron microscopy (cryo-EM), these mitochondria-derived EVs, termed mitovesicles, display an electron dense matrix and a double membrane, a unique property when compared to exosomes and microvesicles isolated from the same tissues, which are single membrane-bound vesicles [2, 8]. These data are in agreement with previous description of the presence of mitochondrial material in EVs [11–14]. While these previous studies investigated the content of heterogeneous pools of EVs, we found that mitochondrial material is uniquely included in one subtype of EVs (mitovesicles) and absent from microvesicles and exosomes. When compared to intracellular mitochondria, mitovesicles differ in size, as mitovesicle diameter is 50–350 nm [2, 8], while the minor mitochondria axis is 200–700 nm and the major axis is several µm in vivo [15]. They also differ in morphology, as mitovesicles lack *cristae* and the intermembrane space is narrower, ~ 6 nm in mitovesicles [2, 8] vs. ~ 20 nm in mitochondria [16]. Furthermore, mitovesicles carry only a restricted, mostly catabolic set of mitochondrial constituents, roughly ~ 24% of the total brain mitochondrial proteome [2], and lack several proteins that are abundant in mitochondria, including TOMM20, mitofusin-2 (MFN2), and the polymerase-γ (POL-γ) subunits [2, 8].

Our mass spectrometry data analysis of the content of mitovesicles in the brain of wild-type mice demonstrated that monoamine oxidase (MAO) type A and B (hereafter MAO-A and MAO-B) are part of the restricted subset of mitochondrial proteins contained in mitovesicles [2]. Intracellularly, MAO isozymes are anchored to the outer mitochondrial membrane where they catalyze the degradation of neuroactive monoamines, including dopamine, serotonin, and noradrenaline, to generate H_2_O_2_ (which, in turn, causes oxidative stress) as a byproduct of the reaction [17]. Thus, MAO-A and MAO-B are at a crossroad between reactive oxygen species accumulation, synaptic regulation, and mitochondrial health, and MAO-B in particular has established roles in neurodegenerative diseases [18], including in AD [19–21].

Given the presence of MAOs in mitovesicles, we hypothesized that these EVs function as neurotransmitter scavengers. In addition, we posited that mitochondrial abnormalities typically found in most neurological conditions [22–27] trigger alterations in mitovesicles, perturbing their activity and contributing to propagation of pathology and possibly to synaptic deficits via mitovesicle MAOs. In this study, we tested these hypotheses in the murine model of DS Ts[Rb(12.17^16^)]2Cje, hereafter called Ts2 [28], an archetype of the interrelationship between mitochondrial dysfunctions, cognitive decline, and mitovesicle abnormalities, given *(i)* the mitochondrial dynamics alterations found in DS, both in humans and in mouse models [22–25, 27], *(ii)* the importance of DS as a model for the pathogenesis of AD, with the progression to AD in DS correlating with mitochondrial and metabolic dysfunctions [25], and *(iii)* findings demonstrating that the same abnormalities in mitovesicle secretion are found in the brains of 12-month-old Ts2 mice and in human DS frontal cortices [2], suggesting that the Ts2 model is an ideal surrogate of the human counterpart to study mitovesicle biology. We found that Ts2 mitovesicles perturb long-term potentiation (LTP) of otherwise normal murine hippocampi, while microvesicles and exosomes isolated from the same brains and mitovesicles isolated from control brains do not impair LTP. We also found that Ts2 mitovesicles carry higher levels of MAO-B when compared to diploid (2N) wild-type controls, and that their electrophysiological effects are MAO-B-dependent. These data show that mitovesicles acquire pathogenic functions under conditions of mitochondrial dysfunction, suggesting that they affect memory formation and the propagation of neuropathology.

## Methods

### Murine strains and experimental design

All mice were purchased from the Jackson Laboratory (Bay Harbor, ME, USA). The B6EiC3Sn-Rb(12.Ts17^16^65Dn)2Cje/CjeDnJ (Ts2, RRID: IMSR_JAX:004850) strain is the result of a spontaneous Robertsonian fusion between the murine chromosome 12 and the small Ts65Dn marker chromosome that carries the distal end of the murine chromosome 16 (orthologous to the human region on chromosome 21 containing the genes triplicated in Down syndrome, including the amyloid-β precursor protein (*App*) gene [28]**)**. Ts2 mice were bred and maintained on a B6EiC3SnF1/J background (2N, RRID: IMSR_JAX:001875) in the Nathan S. Kline Institute for Psychiatric Research animal facility. Colonies were routinely refreshed every third generation with new breeding stocks from Jackson Laboratory to prevent genetic drift. Only *Rd*^wt/wt^ mice (which do not carry a mutation in the retinal degeneration gene) were used. Mitovesicles and other EVs were isolated in all our experiments from the brains of 2N and Ts2 mice when they reached 12 months of age, as described previously [2]. C57BL/6J mice (indicated as wild-type mice in the text, RRID: IMSR_JAX:000664) were bred and maintained in the Columbia University animal facility and used to obtain hippocampal slices for electrophysiology. For both lines, females and males were used in all experiments and compared. No sex differences were found in mitovesicle and mitochondria perturbations caused by trisomy as well as in mitovesicle electrophysiological properties. Accordingly, when applicable, in order to increase statistical power, both males and females were analyzed together, abiding by the National Institutes of Health (NIH, Bethesda, MD, USA) guidelines to reduce to minimum the number of euthanized mice. Cages were randomly allocated for all experiments. All animal procedures were performed following the ‘Animal Research: Reporting In Vivo Experiments’ (ARRIVE) guidelines and the NIH guidelines with approval from the Institutional Animal Care and Use Committees at the Nathan S. Kline Institute for Psychiatric Research and at Columbia University. The characterization of mitovesicles and of other brain EVs complied with the most updated ‘Minimal Information for Studies of Extracellular Vesicles’ (MISEV2023) guidelines [29].

### Human brain tissues and other reagents

Frozen postmortem brain tissues (Brodmann area 9) were a kind gift from Dr. J. Wegiel, head of the Brain and Tissue Bank Department, Department of Developmental Neurobiology, New York State Institute for Basic Research in Developmental Disabilities, Staten Island, NY, USA. Five tissue samples per genotype (2 males and 3 females for diploid controls; 1 male and 4 females for DS; age range: 31- to 68-year-old for 2N; 43- to 60-year-old for DS) were homogenized for Western blotting. 3 tissue samples per genotype (1 male and 2 females for the diploid controls; 1 male and 2 females for DS; age range: 59- to 68-year-old for 2N; 59- to 60-year-old for DS) were used to isolate mitovesicles. Unless specified otherwise, all reagents and equipment were purchased from either Sigma-Aldrich (St. Louis, MO, USA) or Thermo Fisher Scientific (Waltham, MA, USA). The list of the primary antibodies used for Western blot analyses and flow cytometry is provided (Table S1).

### Isolation and manipulation of microvesicles, exosomes, and mitovesicles from the brain extracellular space

EVs were isolated from human and murine brain tissue as described in detail previously [8]. Murine EVs were always isolated from the right hemibrain, as previously described [2, 8]. In short, tissues were treated for 15 min with 20 U/mL papain (Worthington, Lakewood, NJ, USA) in Hibernate A (Brainbits, Springfield, IL, USA) centrifuged at 300*g* for 10 min at 4 °C, filtered with a 40 μm strainer and subsequently with three 0.2 μm surfactant-free cellulose acetate filters (Cat# 431,219, Corning Inc., Corning, NY, USA) [8], differentially centrifuged at 2,000*g* for 10 min, 10,000*g* for 30 min, and 100,000*g* for 70 min (*k*-factor: 207.5, Type 45 Ti rotor, Beckman Coulter, Brea, CA, USA) at 4 °C, and washed in ice-cold phosphate-buffered saline (PBS) to obtain a crude brain EV pellet [3–5]. Brain EV subtypes were separated using a high-resolution iodixanol (OptiPrep™) step-gradient approach [2, 6–9] through which mitovesicles were enriched within the denser part (fraction 8), exosomes in the intermediate part (fractions 4–6), and microvesicles in the lighter part (fractions 1–3) of the gradient. To test the involvement of mitovesicle MAOs in LTP impairment, immediately after the isolation, the same number of mitovesicles was split into three tubes and incubated with either *(i)* 1 µM clorgiline (irreversible MAO-A inhibitor), *(ii)* 1 µM pargyline (irreversible MAO-B inhibitor), or *(iii)* an equivalent volume of dimethyl sulfoxide (DMSO, control) for 10 min at 37 °C. Afterwards, the samples were centrifuged at 100,000*g* (50,000 rpm) in a TLA-55 rotor (*k*-factor: 79.9, Beckman Coulter) for 70 min at 4 °C and the supernatant discarded. Finally, mitovesicles were washed once in PBS to eliminate residual drugs and resuspended in artificial cerebrospinal fluid(aCSF: 124 mM NaCl, 4.4 mM KCl, 1 mM Na_2_HPO_4_, 25 mM NaHCO_3_, 2 mM CaCl_2_, 2 mM MgCl_2_, 10 mM glucose) for electrophysiology.

### Brain homogenization

Murine and human brain tissues were homogenized in 10:1 *v/w* tissue homogenization buffer (0.25 M sucrose, 20 mM Tris-HCl pH 7.4, 1 mM EDTA, 1 mM EGTA) supplemented with protease inhibitors (5 µg/ml leupeptin, 5 µg/ml antipain dihydrochloride, 5 µg/ml pepstatin A, 1 mM phenylmethanesulfonyl fluoride, 1 µM E64) immediately before homogenization. Homogenates were obtained with 20 complete strokes with a Teflon pestle (Wheaton, DWK Life Sciences, Millville, NJ, USA). Aliquots were stored at -80 °C until use.

### Western blot

Murine brain EVs were analyzed by immunoblotting as previously described in detail [8]. For mitovesicle content analyses, equal numbers of mitovesicles were loaded, as quantified by nanotrack analysis (NTA) [2, 8]. To investigate the number of secreted EVs, equal volumes (5 µL) of EVs were loaded. The brain homogenate (BH) analyses were performed on equal protein amounts (5 µg). The commercial primary antibodies used were: mouse monoclonal anti-β-actin (clone 8H10D10, RRID: AB_2242334, Cell Signaling Technology, Danvers, MA, USA), rabbit monoclonal anti-annexin A1 (clone EPR19342, RRID: AB_2890907, Abcam, Cambridge, UK), mouse monoclonal anti-COX-IV (clone mAbcam33985, RRID: AB_879754, Abcam), mouse monoclonal anti-DRP1 (clone 8, RRID: AB_398424, BD Bioscience, San Jose, CA, USA), mouse monoclonal anti-HSC70 (clone B-6, RRID: AB_627761, Santa Cruz Biotechnology, Santa Cruz, CA, USA), rabbit monoclonal anti-MAO-A (clone EPR7101, RRID: AB_11129867, Abcam), rabbit polyclonal anti-MAO-B (RRID: AB_11008931, Novus Biologicals, Littleton, CO, USA), rabbit polyclonal anti-MDH2 (RRID: AB_2878143, Proteintech, Rosemont, IL, USA), rabbit polyclonal anti-MFF (RRID: AB_2142463, Proteintech), mouse monoclonal anti-MFN2 (clone 6A8, RRID: AB_2142629, Abcam), mouse monoclonal anti-NDUFB8 (clone 20E9DH10C12, RRID: AB_2629281, Abcam), rabbit monoclonal anti-phospho-DRP1 (clone D9A1, RRID: AB_11178659, Cell Signaling Technology), rabbit monoclonal anti-POL-γ (clone EPR7296, RRID: AB_11145308, Abcam). The mouse monoclonal anti-APP antibody (clone C1/6.1; RRID: AB_2564648) was a kind gift from Dr. P. Mathews [30]. Sypro Ruby total protein staining was performed as described [8]. All protein bands were acquired with the automated iBright FL1500 imaging system (iBright Analysis Software, RRID: SCR_017632). Densitometry quantification of protein bands was performed through the software ImageJ (version 1.53t; RRID: SCR_003070) [31]. Original, uncropped blots are provided (Fig. S1).

### Electrophysiology

Coronal hippocampal slices (400 μm) were cut with a tissue chopper and transferred to a recording chamber where they were allowed to recover for 2 h. During the recovery period and the recording, slices were maintained at 29 °C and perfused with aCSF. aCSF was bubbled with 95% O_2_ and 5% CO_2_ and perfused at a flow rate of 2 mL/min. Field excitatory post-synaptic potentials (fEPSPs) were measured after stimulation of the Schaffer collateral fibers by a bipolar tungsten electrode placed in the hippocampus at the level of the CA3 and recording at the *stratum radiatum* of CA1 with a glass pipette filled with aCSF. After evaluating the basal synaptic transmission through measurement of the input-output relationship, a 10-minute baseline was recorded every minute at an intensity eliciting a response of approximately 35% of the maximum evoked response. Preparations containing either mitovesicles, microvesicles, or exosomes were diluted in aCSF to a final concentration of 10^8^ EVs/mL, 3 × 10^8^ EVs/mL, or 10 × 10^8^ EVs/mL (as quantified by NTA) and the slices perfused for 20 min prior to tetanization. LTP was induced through a theta-burst stimulation (4 pulses at 100 Hz, with the bursts repeated at 5 Hz and 3 tetani of 10-burst trains administered at 15-second intervals) [32]. Responses were recorded for 2 h after tetanization and measured as fEPSP slope (expressed as percentage of baseline).

### Single-particle flow cytometry

EVs were isolated from the brain of wild-type mice, and the concentration of Fr8 EVs (mitovesicle-enriched), as well as of a 1:1:1 *v/v/v* mixture of fraction 1, 2, and 3 EVs (Fr1-3, microvesicle-enriched, EV-specificity control) quantified by NTA. The volume of PBS containing 1 × 10^10^ EVs was readjusted to a final volume of 30 µL, in order to reach in all cases the final concentration of 3.3 × 10^8^ EVs/µL. EVs were then incubated with either vehicle (secondary only control), 0.158 µg rabbit anti-MAO-A (Abcam Cat# ab126751; RRID: AB_11129867), or 0.025 µg rabbit anti-MAO-B (Novus Cat# NBP1-87493; RRID: AB_11008931) antibodies, mixed well, and left for 1 h at 4 °C with rotation. Subsequently, 0.5 µg donkey anti-rabbit Alexa Fluor 488 secondary antibodies (Thermo Fisher Scientific) were added to all samples and incubated for 30 min at 4 °C with rotation. After staining, sample volumes were brought up to 1 mL using PBS and mixed well. Analyses were conducted on a BigFoot flow cytometer (Thermo Fischer Scientific) using the standard 100 μm nozzle tip at 30 psi (pound-force per square inch). EVs were detected using the 405 nm forward scatter (FSC) small particle detector and the 488 nm side scatter (SSC) laser on a Log_10_ scale. 405 nm (violet) in lieu of classical 488 nm (blue) lasers were used to grant greater resolution of EVs [33]. Alexa Fluor 488 fluorescence was collected using a 488 nm laser with a 507/19 nm bandpass filter. 20,000 EVs were acquired for each experiment. The percentage of positive EVs was determined on the basis of the secondary antibody control gate of each sample (set to 0.8% positivity maximum). All raw metadata, including quality controls such as beads, buffer-only, or EV-only controls are freely accessible at http://flowrepository.org/id/FR-FCM-Z77A. Reporting of EV flow cytometry analyses complied with the MIFlowcyt-EV guidelines (Table S2 and Table S3) [34].

### Other brain mitovesicle analyses

Cryogenic electron microscopy (cryo-EM), NTA, and the monoamine oxidase activity analyses were performed as previously described in detail [2, 8]. To evaluate EV diameter by cryo-EM, the area occupied by each vesicle in each photomicrograph was measured using ImageJ, and the diameter calculated *post-hoc* assuming a circular shape, by the formula d = 2√(A/π), where d is the diameter and A is the area of the EV. NTA was used to measure the hydrodynamic diameter and the number of EVs, as described [2, 8]. Data on mitovesicle number were normalized to the weight of the brain tissue from which EVs were isolated. For PK treatments, mitovesicles isolated from the brain of a 2N mouse were split into 5 tubes prior to the start of the assay. A differential amount of 100 µg/mL PK (Viagen, Cedar Park, TX, USA) resuspended in PBS (or just PBS as a negative control) was added to mitovesicles, and all samples were brought to the same final volume, so that each sample was incubated with either *(i)* no PK (negative control), *(ii)* 10 µg/mL PK, *(iii)* 20 µg/mL PK, *(iv)* 40 µg/mL PK, or *(v)* 40 µg/mL PK supplemented with 0.1% sodium dodecyl sulfate (SDS, positive control). SDS is a strong detergent and was used to solubilize mitovesicle membranes, so that all proteins are accessible to PK digestion, regardless of internal or external location. Mitovesicles were incubated in a water bath at 37 °C for 30 min followed by PK inactivation both chemically (by the addition of protease inhibitors) and thermically (heat-inactivated for 5 min at 95 °C) before preparation of Western blot samples as previously described [8]. The monoamine oxidase assay was performed using a commercial kit (Cat# MAK136, Sigma-Aldrich) according to manufacturer’s instructions with minor adjustments, as described [2]. Briefly, 1 µg mitovesicles isolated either from human or murine brain tissue was incubated or not with 1 µM pargyline (irreversible MAO-B inhibitor) for 10 min at room temperature in a 96-well microplate before addition of the “Master Reaction Mix” provided with the kit. Fluorescence-optimized, dark microplates (Corning) were used to increase signal-to-noise ratio. Plates were read after 2 h at room temperature [2] in a SpectraMax microplate reader (Molecular Devices, San Jose, CA, USA) with excitation/emission wavelengths of 530/585 nm, respectively. An H_2_O_2_ standard curve (provided with the kit) was used to estimate MAO-B units (U) of active enzyme per liter (U definition: 1 U is the amount of enzyme that catalyzes the formation of 1 µmol of H_2_O_2_ in 1 min under the assay conditions) with the formula: MAO-B Activity (U/L) = (FLU_sample_– FLU_sample+pargyline_) / (120_minutes of incubation_ × Slope), where FLUs are the respective raw fluorescence units of the sample with or without pargyline treatment, and the slope is the slope of the standard curve.

### Statistical analyses

The number of mice for each experimental group is indicated in all figure legends as the variable *n*. When numerosity was lower than 15, raw datapoints were provided for each experiment, shown as smaller dots superimposed on the relative bar graph, and histograms reported the mean ± standard error of the mean (SEM) of the distribution. When numerosity was higher than 15, the mean ± SEM for each datapoint or a violin plot were shown instead to improve readability. No datapoints were excluded from any of the analyses. The exact *P* for all comparisons was reported in the relative figure legend. A difference was considered statistically significant when *P* < 0.05 with a 95% confidence interval. Statistical analyses were carried out using either GraphPad Prism (RRID: SCR_002798; version 9.5.0, San Diego, CA, USA) or the Axon pCLAMP software suite (RRID: SCR_011323; version 11, Molecular Devices ). For the cryo-EM quantification of EV diameter, the number of random photomicrographs from three independent isolations considered was in total 25 for Fr1 EVs (microvesicle-enriched, EV-specificity control) and 70 for Fr8 EVs (mitovesicle-enriched), accounting collectively for *(i)* 184 Fr1 EVs isolated from 2N brains (average of 7.4 EVs/field of view), *(ii)* 148 Fr8 EVs isolated from 2N brains (average of 2.1 EVs/field of view), *(iii)* 214 Fr8 EVs isolated from Ts2 brains (average of 3.1 EVs/field of view). The higher number of EVs per field of view in Ts2 vs. 2N mitovesicles is consistent with the higher number of Fr8 EVs in the extracellular space of Ts2 mice, and the overall lower number of EVs per field of view in Fr8 vs. Fr1 EVs was a consequence of the differential number of EVs in these fractions [2, 8].

## Results

### Morphometrical and functional analyses of mitovesicles isolated from the brain of Ts2 mice as compared to diploid littermate controls

**Fig. 1:**
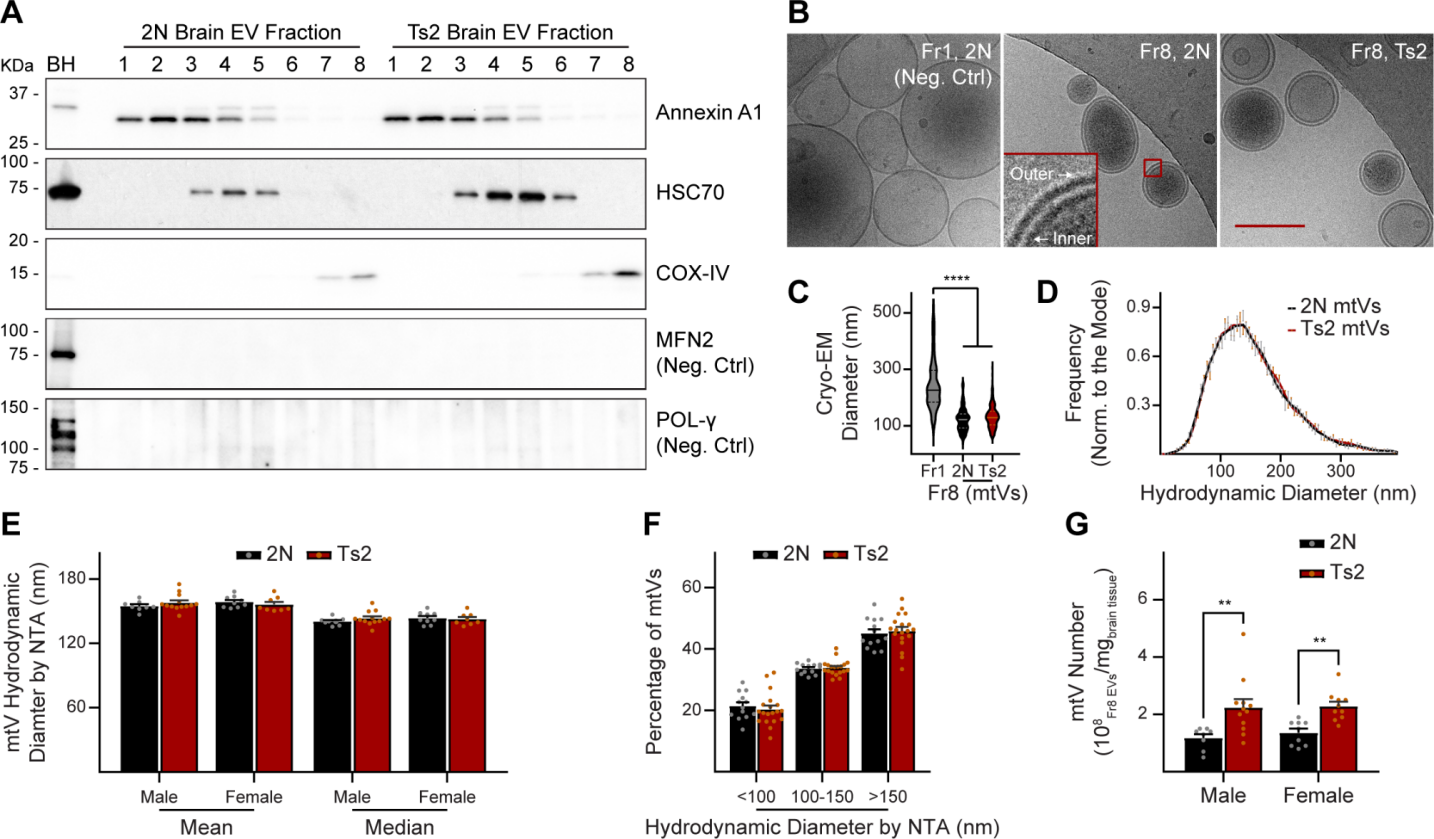
The number of mitovesicles in the brain of Ts2 mice is higher than controls. (**A**) Western blot analyses of EVs isolated from brains of 2N and Ts2 mice. BH: brain homogenate. KDa: kilodaltons. (B-C) Cryo-EM photomicrographs (**B**) and diameter (**C**) of Fr8 EVs (mitovesicle-enriched; ‘mtV’) from 2N (*n* = 148 EVs, 3 mice) and Ts2 (*n* = 214 EVs, 3 mice) brains. Fr1 EVs (microvesicle-enriched; *n* = 184 EVs, 3 mice) are the controls. Scale bar: 200 nm. Data are shown as a violin plot. Fr1 EVs vs. mitovesicles *P* < 0.0001; 2N vs. Ts2 mitovesicles *P* = 0.1713. Kruskal-Wallis *H* test by ranks with Dunn’s multiple comparisons test. (**D**) NTA diameter of mitovesicles from the brain of 2N (*n* = 14) and Ts2 (*n* = 19) mice. Distributions normalized to the mode. Trendline: four-point moving average. Genotype *P* = 0.6271. In (**D-G**), bars are mean ± SEM and the statistical test used was ordinary two-way ANOVA with Bonferroni’s multiple comparisons test. (**E**, **F**) Hydrodynamic size analyses of mitovesicles isolated from 2N (*n* = 8 males, 9 females) and Ts2 (*n* = 13 males, 8 females) brains. In (**E**), for the mean, genotype *P* = 0.8430, sex *P* = 0.6148, genotype X sex *P* = 0.1957, 2N vs. Ts2 within males *P* = 0.5421, 2N vs. Ts2 within females *P* = 0.9016; for the median, genotype *P* = 0.5832, sex *P* = 0.5298, genotype X sex *P* = 0.2900, 2N vs. Ts2 within males *P* = 0.4773, 2N vs. Ts2 within females *P* > 0.9999. In (F), genotype *P* = 0.9970, 2N vs. Ts2 for each bin: *P* > 0.9999. (**G**) Mitovesicle number isolated from 2N (*n* = 8 males, 9 females) and Ts2 (*n* = 12 males, 10 females) brains, quantified by NTA. Genotype *P* < 0.0001, sex *P* = 0.6170, genotype X sex *P* = 0.7450, 2N vs. Ts2 within males *P* = 0.0027, 2N vs. Ts2 within females *P* = 0.0098. ** *P* < 0.01, **** *P* < 0.0001

**Fig. 2:**
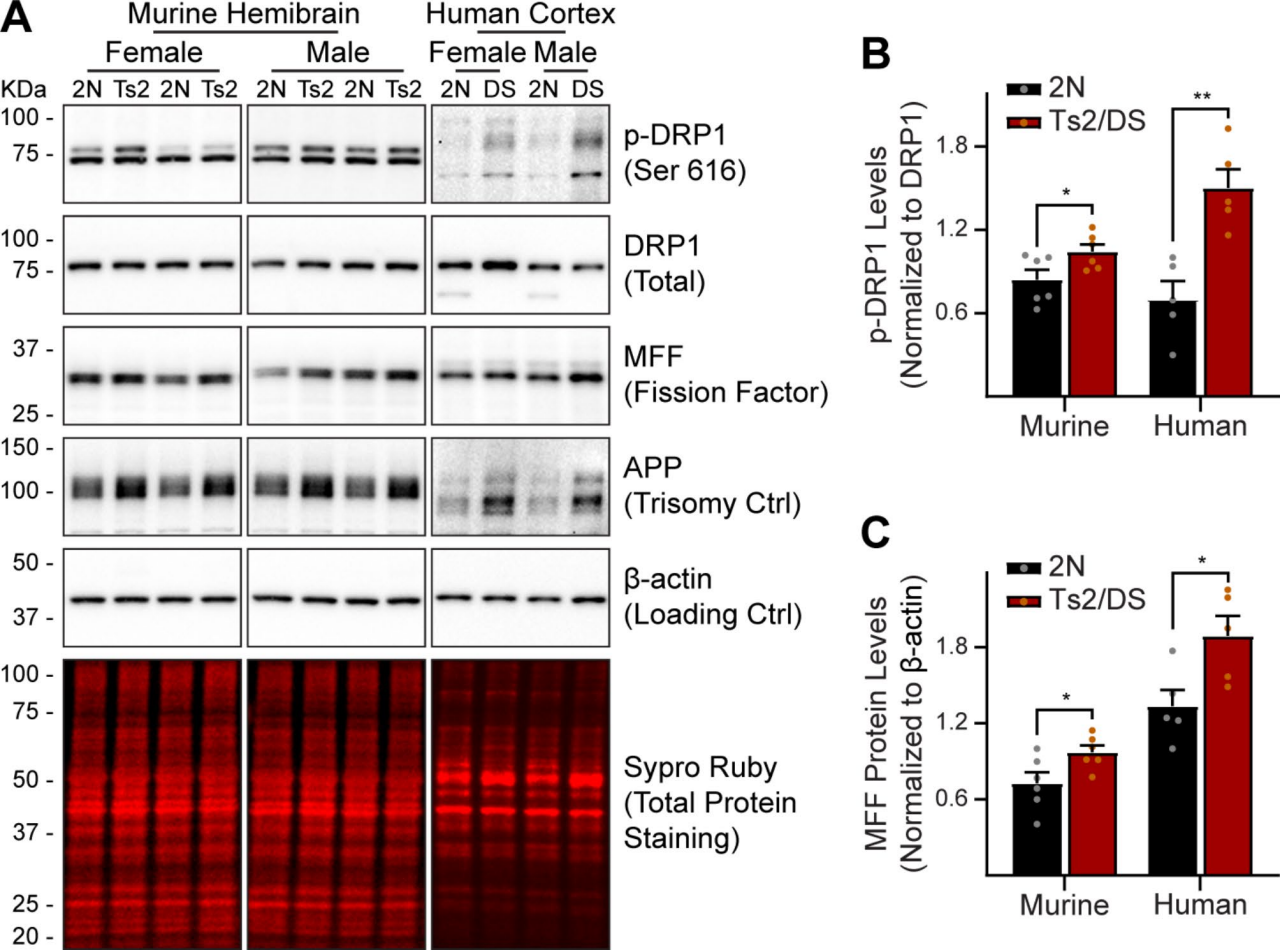
Murine Ts2 and human DS brain homogenates display similar protein alterations associated with mitochondrial fragmentation. (**A-C**) Representative Western blots (**A**) and densitometric quantifications (**B**, **C**) of proteins associated with mitochondrial fission in murine Ts2 brains (*n* = 3 males, 3 females) and human DS frontal cortices (*n* = 5 tissues from different human trisomic donors) as compared to their respective 2N controls (*n* = 3 male, 3 female diploid mice; *n* = 5 tissues from different human diploid donors). Two different pairs of mice for each sex are shown. DRP1 is master regulator of mitochondrial fragmentation (also known as fission) and is activated when phosphorylated at Ser616 (p-DRP1, upper band of the first row). MFF (mitochondrial fission factor) is an outer mitochondrial membrane integral protein and the recruiter of p-DRP1 to the mitochondrial surface. p-DRP1 levels were normalized to total DRP1, while MFF levels were normalized to β-actin. Sypro Ruby (total protein) staining was performed as a loading control. *APP* is one of the triplicated genes in both DS and in Ts2 mice and was used as an internal quality control to confirm the presence of the trisomy. Bars: mean ± SEM. Statistical tests used: two-tailed, unpaired Student’s *t*-test, with a different statistical test for each species, both in (**B**) and in (**C**). In (**B**): 2N vs. Ts2 (murine tissues) *P* = 0.0458; 2N vs. DS (human tissues) *P* = 0.0032. In (**C**): 2N vs. Ts2 (murine tissues) *P* = 0.0374; 2N vs. DS (human tissues) *P* = 0.0255. * *P* < 0.05, ** *P* < 0.01

EVs were isolated from the brain extracellular space of Ts2 mice and 2N littermate controls as previously described [2, 8] and mitovesicles separated from other subtypes of EVs using a high-resolution density gradient (Fig. 1) [2, 8]. Western blotting data revealed the presence of mitovesicle proteins in the EVs found in the fraction number eight of the gradient (Fr8 EVs), including COX-IV, and the concomitant absence of microvesicle-specific proteins (Annexin A1, peaking in Fr1-3 EVs), exosome-specific proteins (HSC70, peaking in Fr4-6 EVs), and proteins found only in mitochondria and in intracellular mitochondria-derived small vesicles but not in mitovesicles (MFN2, POL-γ) [2, 8], indicating purity of the sample (Fig. 1A). As we reported previously [2], the relative abundance of mitovesicle proteins was consistently higher in Ts2 vs. 2N Fr8 EVs (Fig. 1A), in agreement with a higher secretion of mitovesicles by trisomic cells when compared with age-matched diploid controls [2]. Cryo-EM imaging further corroborated the nature of these EVs, demonstrating an enrichment of EVs with a double membrane (typical feature of mitovesicles) [2, 8, 9] in Fr8 as compared with EVs found in other fractions, such as Fr1, that are surrounded by a single membrane (Fig. 1B). Size analyses performed on cryo-EM photomicrographs of Fr8 EVs (Fig. 1C), as well as hydrodynamic diameter quantification of Fr8 EVs using nanotrack analysis (NTA; Fig. 1D-F), demonstrated a size range compatible with mitovesicles for these EVs (50–350 nm) [2, 8, 9]. No size differences were found between 2N and Ts2 mitovesicles in either males or females (Fig. 1C-F), while the number of mitovesicles was higher in Ts2 murine brains as compared to diploid controls, both in males and in females (Fig. 1G), consistent with immunoblotting data. In addition, we confirmed the presence of mitochondrial abnormalities in the brain of Ts2 mice, similar to the molecular changes found in the brain of individuals with DS, including a sex-independent elevation in DS of the phosphorylation of DRP1 at position 616 (p-DRP1) as compared to the respective diploid controls, and higher expression of the mitochondrial fission factor MFF, both markers of mitochondrial fragmentation [9, 35] (Fig. 2). In particular, it was previously shown that higher levels of p-DRP1 correlate with a higher production (and secretion) of mitovesicles [9], consistently with our NTA data (Figs. 1G and 2B). Thus, characterization of Fr8 EVs isolated from mouse brains confirmed the enrichment and purification of intact mitovesicles in this fraction of the gradient. In addition, the isolation of mitovesicles from brains with mitochondrial dysfunction confirmed the presence of alterations in mitovesicle biology resulting in enhanced production and secretion of these EVs [9].

**Fig. 3:**
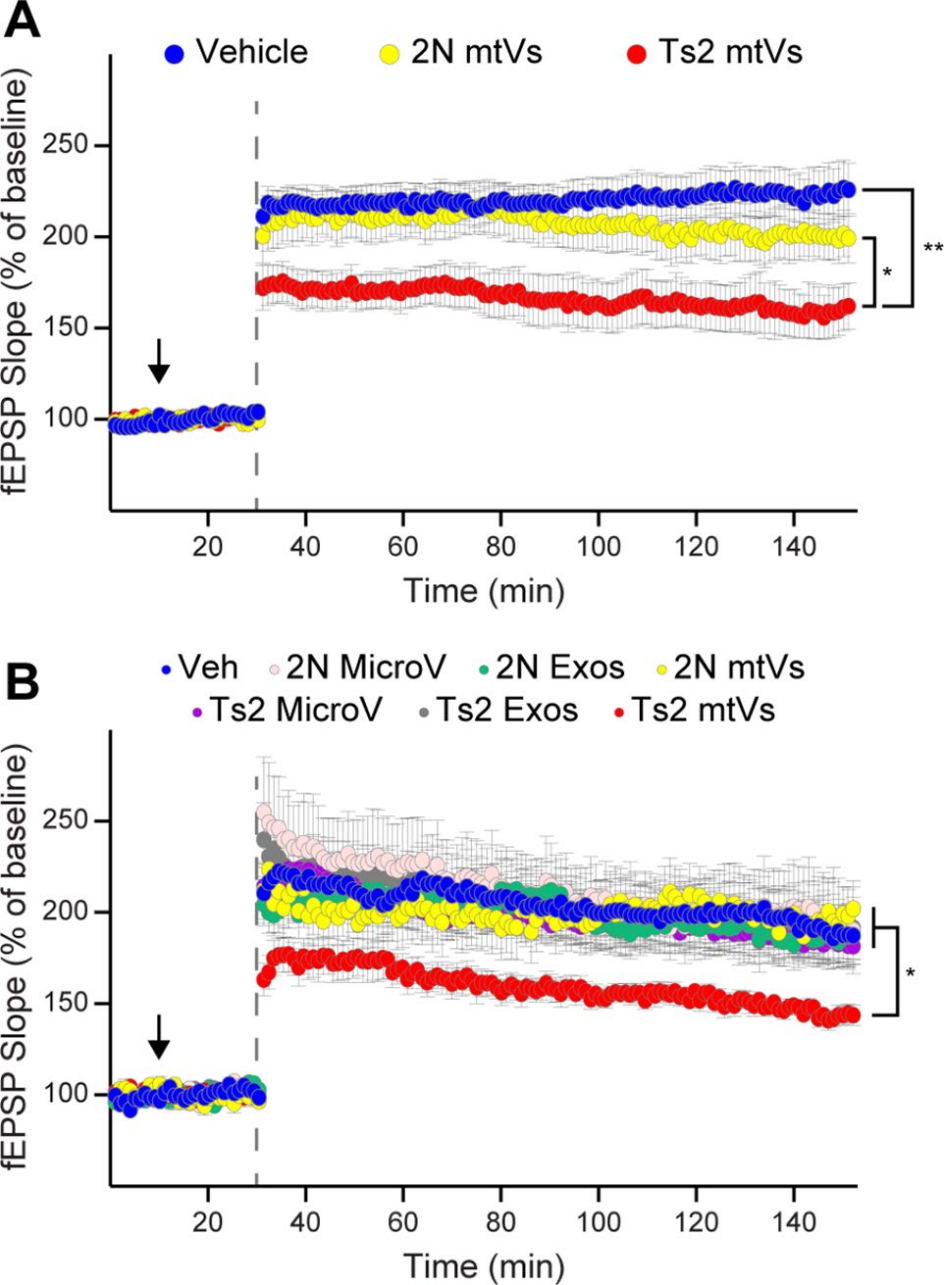
Mitovesicles isolated from the brain of Ts2 mice impair LTP. (**A**, **B**) Induced LTP (dashed lines) 20 min after hippocampal slices were perfused with either aCSF (Vehicle or Veh), or the indicated EV subtype (black arrow; 10^8^ EVs/mL). Fr1 EVs (microvesicle-enriched, MicroV), Fr5 EVs (exosome-enriched, Exos), and Fr8 EVs (mitovesicle-enriched, mtVs), were chosen as the most representative fractions for each EV subtypes [2, 6–8] (see also Fig. 1). Ts2 mitovesicles impaired LTP, both when compared to 2N mitovesicles and to vehicle (**A**). In a separate set of experiments (**B**), microvesicles and exosomes (regardless of the genotype) had no effect on LTP. All tracks report means ± SEM. Two-way ANOVA for repeated measures was used for all comparisons. In (**A**): Vehicle (*n* = 16 slices from 13 mice) vs. Ts2 mitovesicles (*n* = 15 slices from 11 mice) *P* = 0.0022; 2N mitovesicles (*n* = 12 slices from 8 mice) vs. Ts2 mitovesicles *P* = 0.0290; 2N mitovesicles vs. vehicle *P* = 0.4422. In (**B**): 2N microvesicles (*n* = 10 slices from 7 mice) vs. vehicle (*n* = 12 slices from 7 mice) *P* = 0.7784; 2N exosomes (*n* = 13 slices from 8 mice) vs. vehicle *P* = 0.8164; 2N vs. Ts2 microvesicles (*n* = 12 slices from 5 mice) *P* = 0.5685; 2N vs. Ts2 exosomes (*n* = 10 slices from 5 mice) *P* = 0.7867; Ts2 mitovesicles (*n* = 10 slices from 7 mice) vs. vehicle *P* = 0.0275; Ts2 vs. 2N mitovesicles (*n* = 13 slices from 7 mice) *P* = 0.0419; Ts2 mitovesicles vs. Ts2 exosomes *P* = 0.0091; Ts2 mitovesicles vs. Ts2 microvesicles *P* = 0.0039. * *P* < 0.05, ** *P* < 0.01

To test the electrophysiological effects of Ts2 mitovesicles, particularly on pathways that are important for memory consolidation, coronal hippocampal slices were perfused with aCSF (‘vehicle’; ‘veh’ in Fig. 3) alone or containing either 2N or Ts2 mitovesicles (‘mtVs’ in Fig. 3) and LTP was induced at the level of the Schaeffer collateral fibers through a theta-burst stimulation [32]. While 2N mitovesicles did not trigger any effect, Ts2 mitovesicles decreased LTP (Fig. 3A). The effect was quick, as mitovesicles were added 20 min prior to tetanization (Fig. 3, arrows), and the concentration of mitovesicles used (10^8^ EVs/mL) was approximately physiological, the same amount of mitovesicles found in 1 mg of 2N brain tissue and half the amount found in Ts2 brains (1 or 2 ×10^8^ mitovesicles/mg, respectively, Fig. 1G). We next tested whether this effect was mitovesicle-specific or whether it is reproduced by other subtypes of Ts2 EVs (Fig. 3B). Hippocampal slices were perfused with vehicle, 2N or Ts2 microvesicles (Fr1 brain EVs, ‘MicroV’ in Fig. 3B), with 2N or Ts2 exosomes (Fr5 brain EVs, ‘Exos’ in Fig. 3B), or with 2N or Ts2 mitovesicles prior to induction of LTP (10^8^ EVs/mL for each group). Neither microvesicles nor exosomes affected LTP (Fig. 3B), indicating that trisomic mitovesicles are the only type of EVs able to depress hippocampal LTP under these experimental conditions.

### Altered level and activity of mitovesicle MAO-B in Ts2 brains

**Fig. 4:**
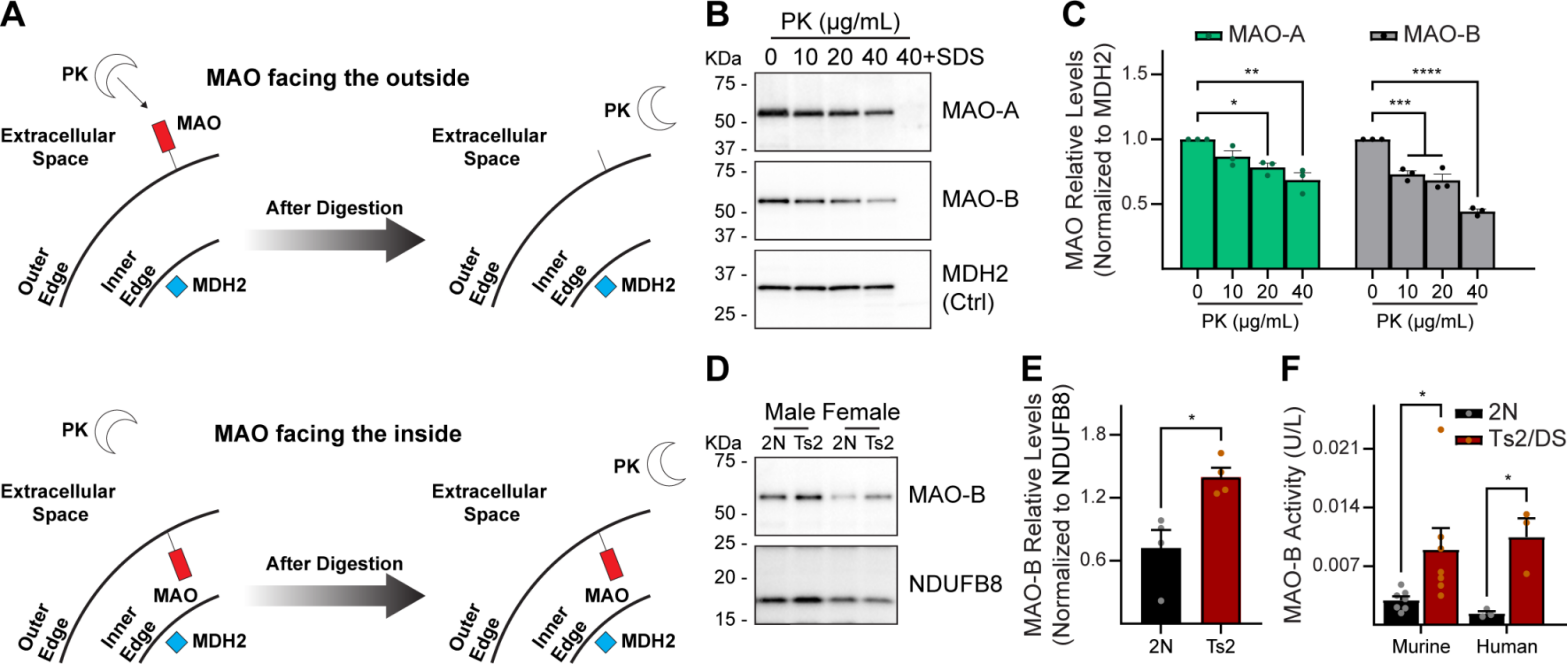
The level and activity of MAO-B are altered in Ts2 mitovesicles. (**A**) Expected results after the incubation of intact mitovesicles with proteinase K (PK). Outer membrane-bound proteins (e.g., MAOs) facing the outside of the EV are cleaved. Proteins that face the inside of the EV and matricial proteins (e.g., MDH2), are shielded by one or two membranes and are not digested. The solubilization of both membranes with a detergent (SDS) causes the degradation of all proteins, regardless of the localization. (**B**, **C**) Representative Western blots (**B**) and densitometric quantifications (**C**) of MAO-A and MAO-B protein levels (normalized to MDH2 levels) in 2N mitovesicles (*n* = 3 mice) upon incubation with PK at increasing concentrations. MDH2: loading control. Last lane: mitovesicles lysed with SDS before PK incubation (positive digestion control). PK effect *P* = 0.0026 for MAO-A and *P* < 0.0001 for MAO-B. For MAO-A: untreated vs. 10 µg/mL PK *P* = 0.1202; untreated vs. 20 µg/mL PK *P* = 0.0122; untreated vs. 40 µg/mL PK *P* = 0.0013. For MAO-B: untreated vs. 10 µg/mL PK *P* = 0.0005; untreated vs. 20 µg/mL PK *P* = 0.0002; untreated vs. 40 µg/mL PK *P* < 0.0001. Ordinary one-way ANOVA with Bonferroni’s multiple comparisons test. In (**C-F**), all bars are mean ± SEM. (**D**, **E**) Representative Western blots (**D**) and densitometric quantifications (**E**) of MAO-B protein levels (normalized to NDUFB8 levels) in equal numbers (quantified by NTA) of mitovesicles isolated from the brain of 2N and Ts2 mice (*n* = 4). NDUFB8 is the loading control to prove similar amount of EVs within each pair. 2N vs. Ts2 mitovesicles *P* = 0.0132. Two-tailed, unpaired Student’s *t*-test. (F) MAO-B enzymatic activity (expressed as U/L) in 1 µg intact mitovesicles isolated from the brain of 2N and Ts2 mice (*n* = 7), or from human DS frontal cortices (*n* = 3) as compared to their respective 2N controls. *P* = 0.0382 and *P* = 0.0150 for murine and human samples, respectively. Two-tailed, unpaired Student’s *t*-test (different test for each species). * *P* < 0.05, ** *P* < 0.01, *** *P* < 0.001, **** *P* < 0.0001

For MAOs to be involved in the LTP impairment induced by Ts2 mitovesicles, these isozymes have to be inserted in the outer mitovesicle membrane and to face the extracellular space. Only in this location is their active site in physical contact with their substrates, as monoamines do not diffuse across biological membranes. Using a proteinase K (PK)-based strategy (Fig. 4A), we analyzed the orientation of MAOs in intact mitovesicles, demonstrating that up to 60% of MAOs are PK-degradable at the highest concentration of PK without solubilizing the double membrane, while MDH2 (a matricial mitovesicle protein involved in the Krebs cycle; negative control) was degraded only when the double membrane was disrupted using a strong detergent (sodium dodecyl sulfate, SDS) (Fig. 4A-C). Although we detected some residual MAOs upon digestion with PK (due either to inefficiency of the protease, steric inhibition of the lipid bilayer, or the presence of a pool of MAOs in the inside of mitovesicles), these data demonstrated that both MAO-A and MAO-B primarily face the external side of brain mitovesicles (Fig. 4A). Moreover, our data showed higher levels and enzymatic activity of MAO-B in Ts2 mitovesicles when compared to diploid littermate controls (Fig. 4D-F).

**Fig. 5:**
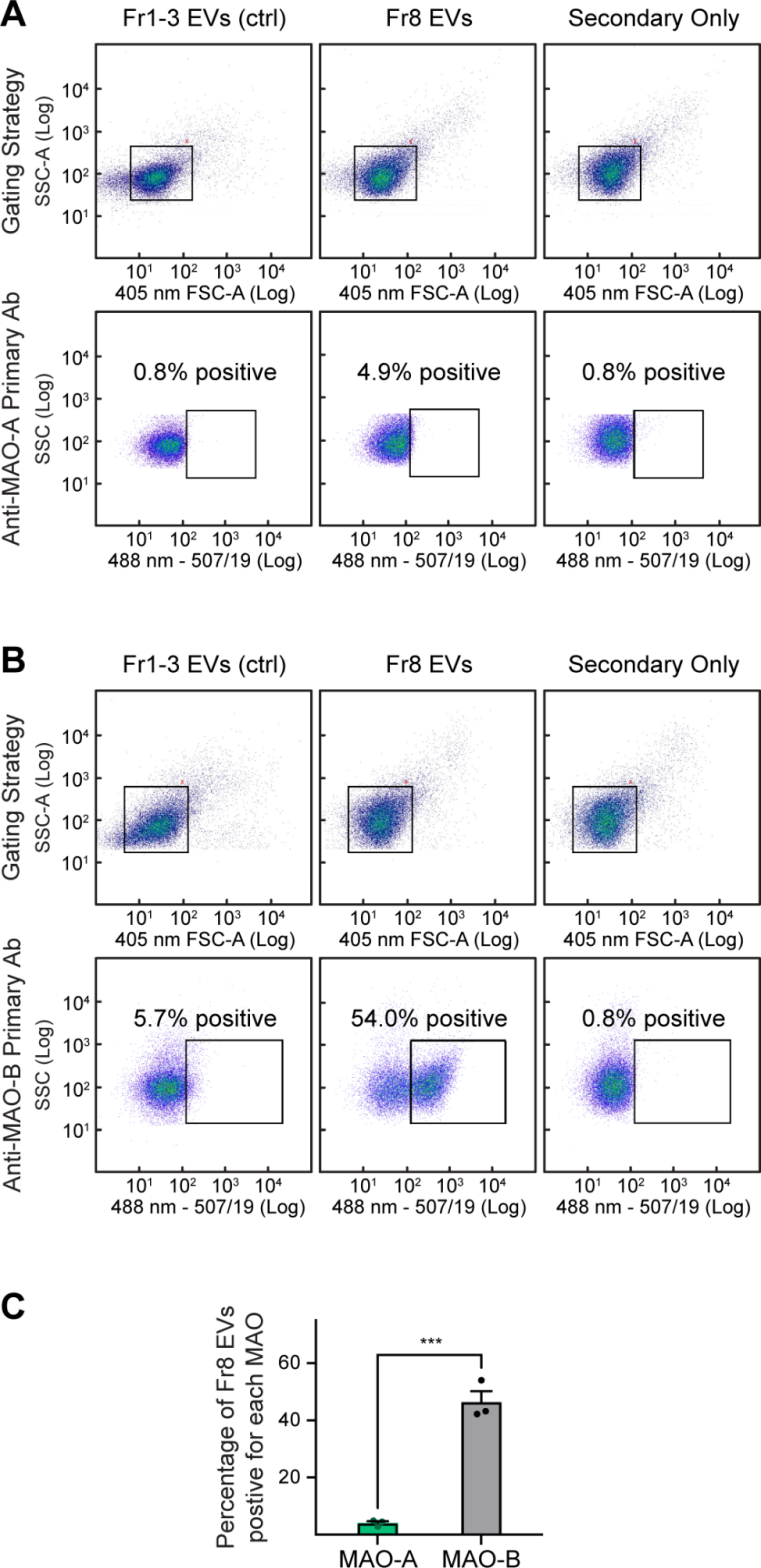
MAO-B-positive mitovesicles are more numerous in the brain than MAO-A-positive mitovesicles. (**A**, **B**) Representative flow cytometry analyses of Fr8 brain EVs (mitovesicle-enriched, second columns) immunolabeled with antibodies against MAO-A (**A**) and MAO-B (**B**). The left columns show the same analyses performed on a 1:1:1 *v/v* mixture of Fr1, Fr2, and Fr3 EVs (microvesicle-enriched) isolated from the same brains (negative control). The right columns show mitovesicles incubated with the secondary antibody only. The gating strategy is shown for all conditions (first rows). Both the y- and the x-axis are in a log_10_ scale. 20,000 EVs were acquired for each experiment. FSC-A: forward scatter– area. SSC-A: side scatter– area. Ab: antibody. (**C**) Percentage of mitovesicles positive for either MAO-A or MAO-B. *n* = 3 mice per group. The percentage of positive EVs was determined on the basis of the secondary antibody control gate of each sample (set to 0.8% maximum). Bars: mean ± SEM. Statistical test used: two-tailed, unpaired Student’s *t*-test. MAO-A vs. MAO-B, *P* = 0.0004. *** *P* < 0.001

In the brain, the expression profiles of MAOs do not overlap: MAO-B displays primarily a broad, pan-astrocytic expression under physiological conditions [18, 21, 36], while MAO-A is found only in monoaminergic neurons [37]. In addition, MAO-B expression can be detected in cortical and hippocampal pyramidal neurons in AD [20]. This suggests that each mitovesicle does not carry both MAOs and is either positive for MAO-B (astrocytic and pyramidal mitovesicles) or for MAO-A (dopaminergic, noradrenergic, and serotoninergic neuronal mitovesicles). To analyze the relative proportion of MAO-B- vs. MAO-A-positive mitovesicles, we used a flow cytometry-based strategy on single mitovesicles (Fig. 5). We first demonstrated that both MAO-A (Fig. 5A) and MAO-B (Fig. 5B) were detected in intact mitovesicles, but not in other types of brain EVs, further reinforcing the conclusion that these proteins are specific components of the mitovesicle outer membrane (Fig. 5A, B). Quantification showed that while 46.5 ± 3.8% (mean ± SEM) of the mitovesicles isolated from a whole hemibrain were positive for MAO-B, only 4.1 ± 0.6% (mean ± SEM) were positive for MAO-A (Fig. 5C), consistent with the relative abundance in the brain of the cells of origin: astrocytes and pyramidal neurons are abundant [38], while monoaminergic neurons comprise only a small fraction of the neurons. As the vast majority of MAO-positive mitovesicles contained MAO-B and not MAO-A, the following analyses focused mostly on MAO-B. We found that MAO-B levels were higher in Ts2 mitovesicles as compared to 2N controls (Fig. 4D, E), indicating either an imbalance in the proportion of MAO-B-positive vs. MAO-B-negative mitovesicles in Ts2 brains or a higher loading of MAO-B per mitovesicle. We next quantified the enzymatic activity of MAO-B in intact mitovesicles upon incubation of these EVs in vitro with tyramine, a monoamine substrate of MAOs that does not cross biological membranes due to its polarity [39]. In agreement with the immunoblotting data, equal numbers of Ts2 mitovesicles degraded more tyramine via MAO-B than 2N controls (Fig. 4F). The same effect was observed when human mitovesicles isolated from the frontal cortex of individuals with DS were evaluated and compared to age-matched diploid individuals devoid of neuropathology (Fig. 4F).

Altogether, these data demonstrated that MAO-B is an enzymatically active component of brain mitovesicles, and that these EVs are able to actively degrade monoamines in a cell-free system. MAO-B orientation (Fig. 4A-C), levels (Fig. 4D, E), and activity (Fig. 4F) suggest that this protein has a role in the LTP impairment induced by Ts2 (but not 2N) mitovesicles (Fig. 1) by changing monoamine homeostasis in otherwise normal hippocampi.

### Role of MAO-B in mitovesicle-induced LTP impairments

**Fig. 6:**
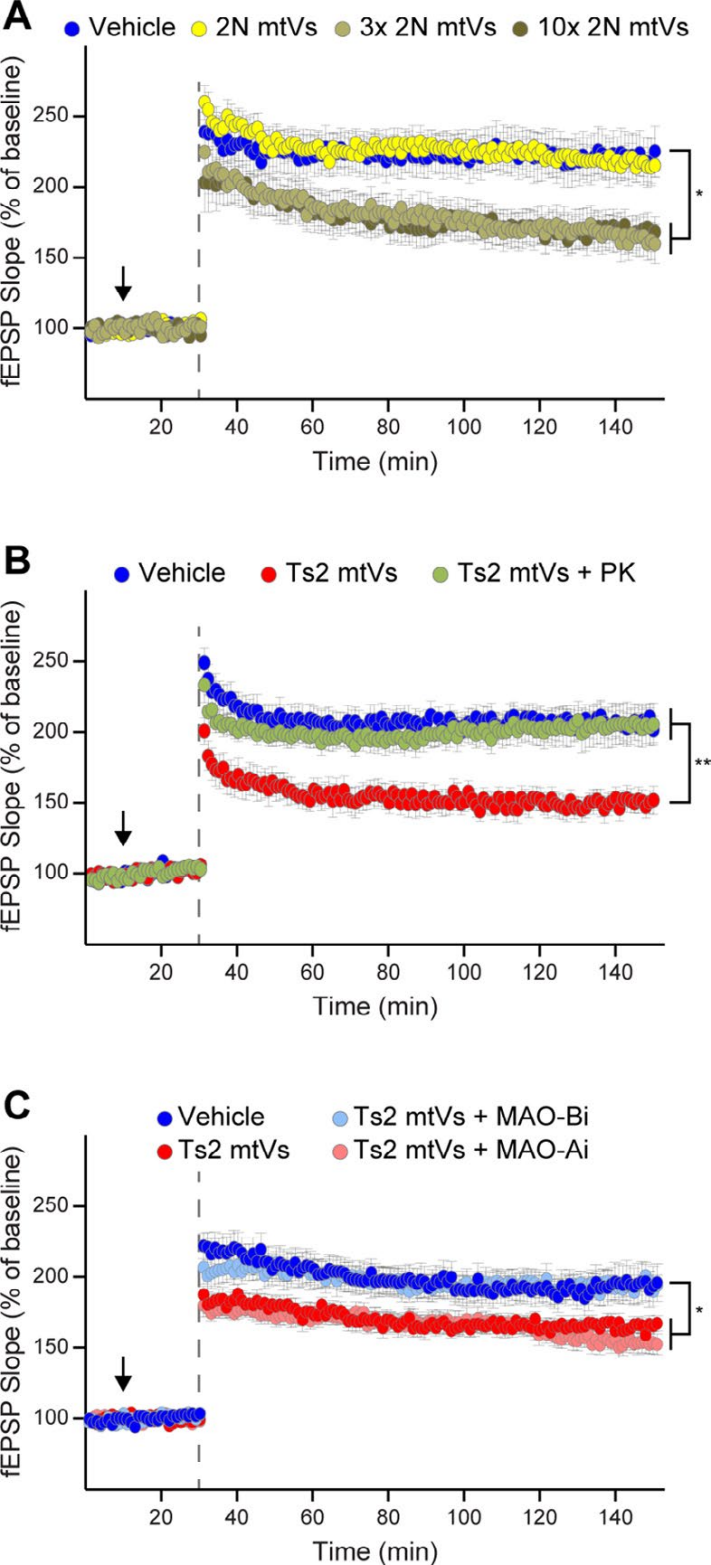
The LTP impairment induced by Ts2 mitovesicles is MAO-B-dependent. (**A-C**) Induced LTP (dashed lines) 20 min after hippocampal slices were perfused with either aCSF (Vehicle) or Fr8 EVs (mitovesicle-enriched, mtVs), as indicated in the figure. In (**A**), slices were treated or not with increasing doses of 2N mitovesicles. In (**B**), Ts2 mitovesicles were pretreated or not with PK (‘PK-shaving’) before addition to the hippocampal slices. In (**C**), mitovesicles were pretreated or not with 1 µM clorgiline (irreversible MAO-A inhibitor, MAO-Ai) or 1 µM pargyline (irreversible MAO-B inhibitor, MAO-Bi). LTP was restored when Ts2 mitovesicles were pretreated with MAO-Bi but not with MAO-Ai. All tracks report means ± SEM. Two-way ANOVA for repeated measures was used for all comparisons. In (**A**): Vehicle (*n* = 23 slices from 14 mice) vs. 10^8^ (*n* = 14 slices from 9 mice) 2N mitovesicles *P* = 0.9342; Vehicle vs. 3 × 10^8^ (*n* = 17 slices from 10 mice) 2N mitovesicles *P* = 0.0330; Vehicle vs. 10 × 10^8^ (*n* = 13 slices from 8 mice) 2N mitovesicles *P* = 0.0423. In (**B**): Vehicle (*n* = 16 slices from 9 mice) vs. Ts2 mitovesicles (*n* = 11 slices from 5 mice) *P* = 0.0007; Ts2 mitovesicles vs. Ts2 mitovesicles + PK (*n* = 14 slices from 9 mice) *P* = 0.0021. In (**C**): Ts2 mitovesicles (*n* = 15 slices from 10 mice) vs. Ts2 mitovesicles + MAO-Bi (*n* = 17 slices from 10 mice) *P* = 0.0150; Ts2 mitovesicles vs. vehicle (*n* = 14 slices from 8 mice) *P* = 0.0158; Ts2 mitovesicles vs. Ts2 mitovesicles + MAO-Ai (*n* = 17 slices from 12 mice) *P* = 0.6630; vehicle vs. Ts2 mitovesicles + MAO-Ai: *P* = 0.0076. * *P* < 0.05, ** *P* < 0.01

Enzymatic data showed that MAO-B activity is three times higher in Ts2 mitovesicles when compared to 2N (Fig. 4F). In order to determine the effect of a higher dose of mitovesicle MAO-B, we incubated hippocampal slices with a three-times higher number of mitovesicles isolated from 2N diploid brains (3 × 10^8^ EVs/mL), demonstrating that this higher number of 2N mitovesicles is sufficient to elicit an alteration in LTP (Fig. 6A). Further increasing the dose to ten times the physiological levels did not show a stronger impairment (Fig. 6A), indicating a plateauing effect. In addition, pretreating Ts2 mitovesicles with 40 µg/mL PK, and therefore reducing the level of MAO-B to less than 50% (Fig. 4C), blocked their electrophysiological activity on LTP (Fig. 6B). Lastly, to analyze the specific role of MAO-B in LTP regulation, equal numbers of Ts2 brain mitovesicles were either (*i)* untreated, (*ii)* pretreated in vitro for 10 min with 1 µM clorgiline (a MAO-A-specific irreversible inhibitor, MAO-Ai), or (*iii)* pretreated with 1 µM pargyline (a MAO-B-specific irreversible inhibitor, MAO-Bi). The residual drug/medium was washed away, and 10^8^ EVs/mL EVs used to perfuse hippocampal slices prior to LTP induction, following the procedure described above (Fig. 6C). Consistent with flow cytometry data demonstrating that MAO-A-positive mitovesicles are only a numerically minor subtype of the total mitovesicle population when these EVs are isolated from a whole hemibrain (Fig. 5), MAO-Ai-pretreated and untreated Ts2 mitovesicles impaired LTP to the same extent (Fig. 6C), suggesting that MAO-A is not a major contributor to the LTP depression induced by Ts2 mitovesicles. Conversely, MAO-Bi-pretreated Ts2 mitovesicles lost the ability to impair LTP, restoring the field excitatory post-synaptic potentials (fEPSPs) to physiological levels upon stimulation (Fig. 6C). These data revealed that the electrophysiological effect of Ts2 mitovesicles was dependent on the enzymatic activity of MAO-B.

## Discussion

In this study we demonstrate that brain cells secrete a higher number (Fig. 1G) of mitovesicles containing more MAO-B (Fig. 4) in DS when compared to 2N controls, causing LTP depression (Figs. 3 and 6). Mitovesicle effects on LTP were quick (20 min) suggesting extracellular enzymatic activity of MAO-B. However, given the lack of knowledge about the cell biology driving mitovesicle production, uptake, and cell fate, we cannot rule out intracellular activity of mitovesicles following internalization by recipient neurons. DS is caused by triplication of the whole chromosome 21 or parts of it, including *App* and genes that are critical for mitochondrial homeostasis [40]. Most individuals with DS that reach 50 years of age develop AD [41] and show phenotypical symptoms of dementia, including an age-dependent buildup of amyloid-β plaques, neurofibrillary tau tangles, cholinergic neuronal loss in the basal forebrain, cognitive decline, hypometabolism, and mitochondrial alterations [22]. An *in vitro* study has shown mitochondrial dysfunctions in fibroblasts derived from 5-month-old individuals with DS [23]. In addition, metabolomic analyses of biofluids of individuals with DS revealed mitochondrial alterations throughout their lifespan, far preceding the onset of dementia [24]. The progression to AD in DS correlates with mitochondrial and metabolic abnormalities [25]. Moreover, in a mouse model of β-amyloidosis, the concomitant presence of both the overexpression of *App* with the London mutation and mitochondrial DNA (mtDNA) mutations promoted the deposition of higher levels ofamyloid-β plaques when compared to the same model without mtDNA alterations [42]. Conversely, the transfer of exogenous fully energized mitochondria into 5XFAD mice ameliorated brain mitochondrial respiration but also cognitive impairment and amyloid-β burden [43]. Altogether, these data suggest synergism between APP/amyloid-β imbalance and mitochondrial damage in causing neurodegeneration with age, and imply, at least partially, a causative role for mitochondria dynamics in the insurgence of dementia-like manifestations.

Among several mitochondrial components that are altered in AD and in people with DS, MAO-B attracted attention because of its role in the regulation of neurotransmission. A large body of evidence links MAO-B activity and monoamine levels to phenotypic manifestations of AD in DS [44]. MAO-B (but not MAO-A) activity is elevated in the hippocampus of AD patients [45], and the levels of several neuroamines (noradrenaline, serotonin, taurine, and dopamine), but not of other common neurotransmitters, such as glutamate, are reduced in the brain of people with DS [46, 47], consistent with an elevation in brain MAO activity in DS. Likewise, in the Ts65Dn murine model of DS, hippocampal serotonin is lower as compared to age-matched 2N controls [48], mimicking human data. In addition, MAO-A/B double knock-out mice exhibit abnormally high hippocampal LTP [49], a direct connection between MAO activity and LTP. Our data demonstrate that mitovesicles are able to stimulate the same electrophysiological alterations, including LTP depression, that are found in AD and in adults with DS in otherwise normal murine hippocampi, in the absence of amyloid-β plaques, tau tangles, or alterations in the processing of APP within the recipient tissue. In addition, although the donor mice used in our experiments (Ts2 mice) carry one extra copy of the rodent *App* gene, human amyloid-β (Aβ) peptides are not associated with their brain EVs, differently from what we described previously for the human *App* overexpressing Tg2576 mice [3, 50]. Rodent Aβ differs when compared to the human counterpart at residues 5, 10 and 13, producing a shorter, non-amyloidogenic cleavage product when processed by murine BACE1 (Aβ_11−40/42_) instead of the human longer Aβ_1–40/42_ peptides [51]. Accordingly, while several mammals, including primates, dogs, and polar bears, develop Aβ oligomers and fibrils with age [52], rodents fail to produce Aβ deposits at any age unless the human *App* gene is exogenously overexpressed, and therefore Aβ fibrils and oligomers are absent in murine lines in which only the rodent gene is upregulated, such as DS murine models and when the *App* rodent gene is upregulated by 5-fold [53–55]. Ts2 mice are no exception, and do not develop Aβ pathology, although they show hippocampal LTP alterations [56]. These data indicate that the electrophysiological impairments in Ts2 brains are largely Aβ-independent.

Thus, mitovesicles arise as active players of the extracellular milieu in the pathophysiology of AD in DS, and as novel connectors of altered pathways typically studied as separate deficits, including mitochondrial dysfunction, EV alterations, spread of pathology, and memory loss via LTP impairment.

## Conclusions

Mitochondrial damage is found in most, if not all, brain diseases, and monoamine metabolism is also crucial for a plethora of conditions unrelated to dementia, such depression, mood disorders, cocaine addiction, and Parkinson’s disease. We previously reported a link between mitochondrial abnormalities and mitovesicle alterations in several conditions that are not linked to AD nor to DS, including chronic cocaine exposure [9]. Therefore, this study may represent an initial platform for broader investigations, aimed to fully understand how mitochondrial alterations impact the extracellular environment and how mitovesicles perturb the brain in potentially all conditions characterized by mitochondrial dysfunction. Our data imply that a novel approach should be undertaken to achieve this goal, shifting from the classical reductionist approach in which the effects of mitochondria abnormalities are studied in the same cells where damaged mitochondria reside to a more holistic point of view in which focal intracellular alterations altering mitochondrial function can impact the homeostasis of distant cells via mitovesicles.

### Electronic supplementary material

Below is the link to the electronic supplementary material.

**Fig. S1. Unprocessed/uncropped Western blot data**. Red boxes surround the area shown in the relative main figure. Arrowheads indicate the remaining signal from previous blotting of the same membrane with different antibodies. KDa: kilodaltons.

**Table S1**. List of primary antibodies used for Western blotting and flow cytometry.

**Table S2**. MIFlowCyt Framework.

**Table S3**. MIFlowCyt Checklist.

## Data Availability

All data generated or analyzed during this study are included in this published article [and its supplementary information files], or available from the corresponding author on reasonable request. Flow cytometry data are accessible at http://flowrepository.org/id/FR-FCM-Z77A.

## Abbreviations

2N: diploid
Aβ: amyloid-β
aCSF: artificial cerebrospinal fluid
AD: Alzheimer’s disease
APP: amyloid-β precursor protein
ANOVA: analysis of variance
BH: brain homogenate
COX-IV: cytochrome c oxidase subunit 4
cryo-EM: cryogenic electron microscopy
DMSO: dimethyl sulfoxide
DRP1: Dynamin-related protein 1
DS: Down syndrome
EM: electron microscopy
EV: extracellular vesicle
Exos: exosome
fEPSP: field excitatory post-synaptic potentials
Fr: brain EV density fraction
FSC: forward scatter
HSC70: Heat shock cognate 71 kDa protein
LTP: long-term potentiation
MAO: monoamine oxidase
MAO-A: monoamine oxidase type A
MAO-Ai: monoamine oxidase type A irreversible inhibitor (clorgiline)
MAO-B: monoamine oxidase type B
MAO-Bi: monoamine oxidase type B irreversible inhibitor (pargyline)
MDH2: malate dehydrogenase isoform 2 (mitochondrial)
MFF: mitochondrial fission factor
MFN2: mitofusin 2
MicroV: microvesicle
miRNA: microRNA
mtDNA: mitochondrial DNA
mtV: mitovesicle
NDUFB8: NADH dehydrogenase [ubiquinone] 1 beta subcomplex subunit 8
NTA: nanotrack analysis
PBS: phosphate buffered saline
PDH-E1α: pyruvate dehydrogenase E1 component subunit alpha
p-DRP1: phosphorylated DRP1 (Serine 616)
PK: proteinase K
POL-γ: polymerase-γ (mitochondrial)
psi: pound-force per square inch
SDS: sodium dodecyl sulfate
SEM: standard error of the mean
SSC: side scatter
Ts2: Ts[Rb(12.17^16^)]2Cje mice
VDAC: voltage-dependent anion channel
