# Supplementary figures and images for: Mitovesicles secreted into the extracellular space of brains with mitochondrial dysfunction impair synaptic plasticity
Source: Mol Neurodegener. 2024 Apr 14;19:34. doi: 10.1186/s13024-024-00721-z (PMC11017499; doi:10.1186/s13024-024-00721-z)

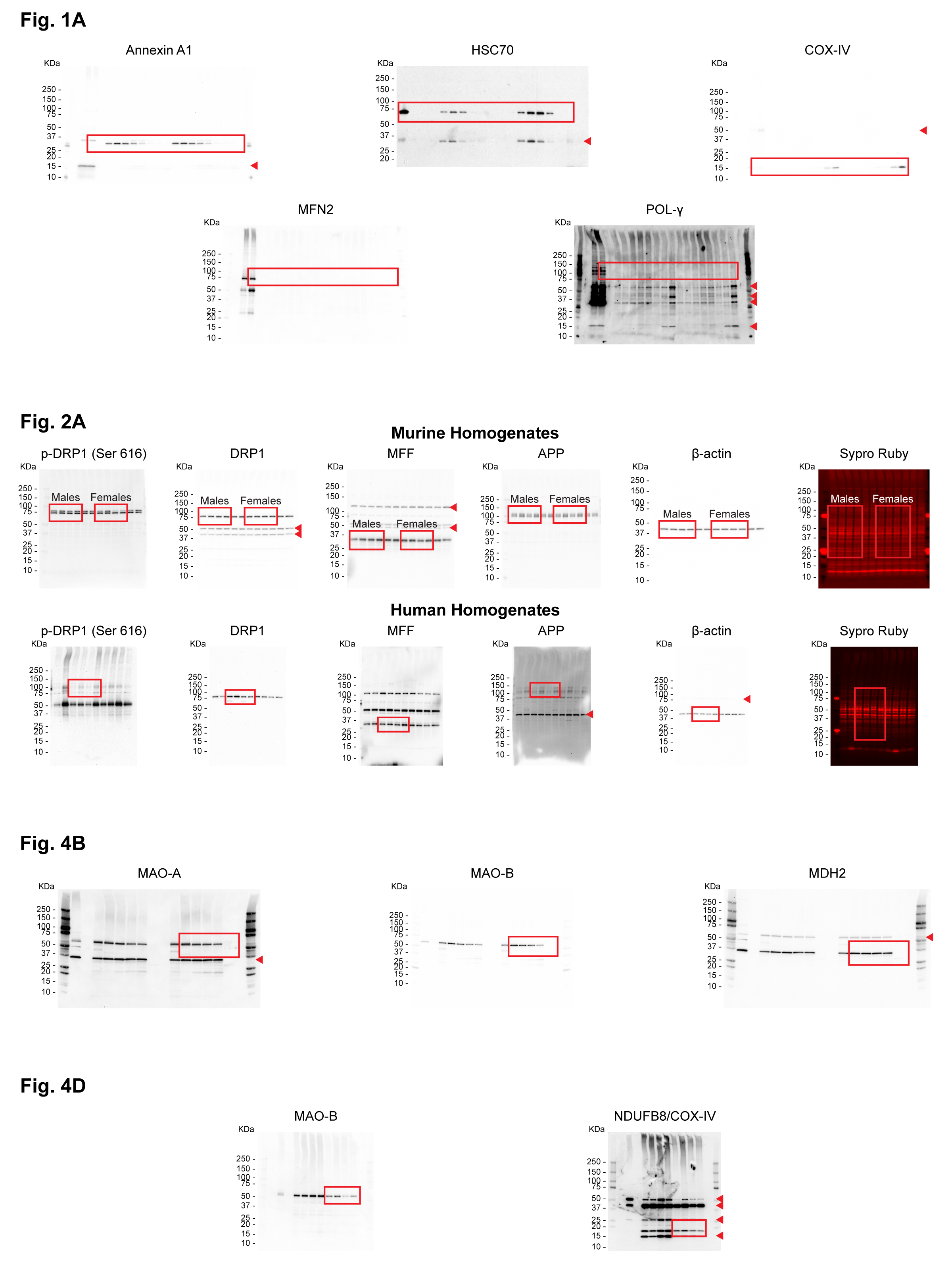

Supplement: Supplementary file 1 — Fig. S1. Unprocessed/uncropped Western blot data. Red boxes surround the area shown in the relative main figure. Arrowheads indicate the remaining signal from previous blotting of the same membrane with different antibodies. KDa: kilodaltons. [file 13024_2024_721_MOESM1_ESM.tif]
