# Supplementary material for: Mitovesicles secreted into the extracellular space of brains with mitochondrial dysfunction impair synaptic plasticity
Source: Mol Neurodegener. 2024 Apr 14;19:34. doi: 10.1186/s13024-024-00721-z (PMC11017499; doi:10.1186/s13024-024-00721-z)
Supplement: Supplementary file 4 — Table S3. MIFlowCyt Checklist. [file 13024_2024_721_MOESM4_ESM.doc]

| **Requirement** | **Please Include Requested Information** |
| --- | --- |
| 1.1. Purpose | The aim of the flow cytometry performed in the study is to determine the amount of MAO-A and MAO-B on the single mitovesicles derived from adult murine brains. |
| 1.2. Keywords | Mitovesicles, MAO-A, MAO-B, and Immunolabeling |
| 1.3. Experiment variables | Microvesicle-enriched EVs, Mitovesicle-enriched EVs, Antibody incubation (primary + secondary, secondary antibody alone or no antibodies) |
| 1.4. Organization name and address | Nathan S. Kline Institute,  140 Old Orangeburg Rd, Orangeburg, NY 10962 USA |
| 1.5. Primary contact name and email address | Name: Christopher Bare  Email address: christopher.bare@nki.rfmh.org |
| 1.6. Date or time period of experiment | May 10^th^ 2022 - June 3^rd^ 2022. |
| 1.7. Conclusions | MAO-B-positive mitovesicles are more numerous than MAO-A-positive mitovesicles in the mouse brain. |
| 1.8. Quality control measures | The flow cytometer was calibrated by using Megamix-Plus FSC (cat # 01077) and Megamix-Plus SSC (cat # 01078) beads (Stago Diagnostica) to detect small extracellular vesicles. The machine underwent the built-in QC procedure before the use. |
| 2.1.1.1. (2.1.2.1., 2.1.3.1.) Sample description | The samples are extracellular vesicles (EVs) resuspended in PBS |
| 2.1.1.2. Biological sample source description | The EVs were isolated from rodent brains. |
| 2.1.1.3. Biological sample source organism description | 12-month-old C57BL/6 mice. |
| 2.1.2.2. Environmental sample location | N/A |
| 2.3. Sample treatment description | The EVs were incubated with primary antibodies and subsequently fluorescence-conjugated secondary antibodies or only with fluorescence-conjugated secondary antibodies as controls. EVs without any treatment were also used. |
| 2.4. Fluorescence reagent(s) description | Donkey anti-rabbit conjugated to Alexa Fluor 488 as a secondary antibody (Thermo Fisher Scientific, Cat# A21206) |
| 3.1. Instrument manufacturer | Invitrogen/Thermo Fisher Scientific |
| 3.2. Instrument model | Bigfoot |
| 3.3. Instrument configuration and settings | Purchased as catalog configuration PL00303, 5 lasers: 488nm (125mW, 5 fluorescent and 4 light scatter detectors), 405nm (100mW, 7 fluorescence and 1 scatter detector), 349nm (100mW 7 fluorescence detectors), 561nm (120mW 7 fluorescence detectors), and 640nm (100mW, 4 fluorescence detectors). |
| 4.1. List-mode data files | *We recommend all authors to submit their data files to [http://flowrepository.org](http://flowrepository.org/) and to make them available for the peer-review process. If you have done so, please let us know by inserting the following codes (replace the red text):  1) The link for peer-review process:  http://flowrepository.org/id/FR-FCM-Z77A. This link will only be shared with reviewers of your manuscript.  2) The repository identifier:  http://flowrepository.org/id/FR-FCM-Z77A. This link will be made publicly accessible after the paper is published. |
| 4.2. Compensation description | The flow cytometry was performed to detect single-stained brain EVs. Thus, compensation was not used. |
| 4.3. Data transformation details | N/A |
| 4.4.1. Gate description | The EV populations detected by 488 nm SSC and 405 nm FSC, excluding debris, were gated to obtain Alexa 488 fluorescence positive EV particles. |
| 4.4.2. Gate statistics | The percentage of positive EVs was determined on the basis of the secondary antibody control gate of each sample. |
| 4.4.3. Gate boundaries | The secondary antibody control gate was set to 0.8% maximum. |
